# Evaluating the Quality of Life of 231 Children With Primary Nephrotic Syndrome and Assessing Parental Awareness of the Disease

**DOI:** 10.3389/fped.2021.745444

**Published:** 2021-12-02

**Authors:** Na Li, Jia Hao, Tong Fu, Yue Du

**Affiliations:** Department of Pediatrics, Shengjing Hospital of China Medical University, Shenyang, China

**Keywords:** primary nephrotic syndrome, quality of life, questionnaire survey, disease awareness, children

## Abstract

**Objective:** This study aims to investigate the quality of life of children with primary nephrotic syndrome (PNS), assess their parents' disease awareness, and provide a basis for the comprehensive management of children with PNS.

**Methods:** A total of 231 children with PNS who were hospitalized in the Department of Pediatric Renal Rheumatology and Immunology in the ShengJing Hospital of the China Medical University from March 2019 to October 2020 were selected as the study subjects. The subjects and their parents were surveyed via a disease education and communication WeChat group and online questionnaire to investigate the children's quality of life, the needs of the parents, and their knowledge related to the disease.

**Results:** In 93.51% of cases, the child's quality of life was affected, with mild to moderate effects being the most frequent (90.47%). The lowest overall quality of life scores were recorded for children who had been diagnosed 1–3 year prior to inclusion in the study, and the scores plateaued thereafter. On the physical functioning scale, the longer the illness, the greater the physical impact, with children typically experiencing pain and fatigue. The children generally scored low on the emotional functioning scale, exhibiting sleep disturbances for up to 5 years and worrying about accidents. The children's average score on the social functioning scale was high, with males achieving significantly higher scores (69.61 ± 25.42) than females (62.30 ± 27.51), and more than one-third of the children experiencing problems getting along with other teenagers and making friends. The primary problems expressed by parents were anxiety (59%), sadness (44%), fear (43%), and depression (40%), and several parents indicated that they struggled with issues of self-blame.

**Conclusion:** PNS impacts the physical and psychological wellbeing of children suffering from the condition, significantly reduces their quality of life, and negatively impacts the psychological wellbeing of their parents. Therefore, children with PNS and their families need integrated management by doctors, nurses, dieticians, psychotherapists, educational institutions, and social stakeholders to improve their quality of life.

## Introduction

Primary nephrotic syndrome (PNS) accounts for approximately 90% of all nephrotic syndromes in pediatric patients and is one of the most common glomerular diseases found in children. PNS is a prolonged and recurrent condition requiring long-term therapy with steroids and other immunosuppressive agents, and children are primarily cared for at home, rather than in the hospital. Previous studies have shown that chronic diseases in children have a significant negative impact on growth, involvement in social activities, psychological wellbeing, and other aspects related to quality of life (1–3). PNS is a disease with a long duration and frequent recurrences, with a diverse range of causes that vary from person to person. Therefore, parents need adequate knowledge of the disease. Some parents have misconceptions about steroid therapies and diet, which reduces treatment compliance and makes standardized treatment difficult.

The present study aims to identify physical, psychological, and social problems in children with PNS and assess the knowledge requirements of their parents. We anticipate that this data will provide a valuable point of reference for providing targeted scientific education for children and parents to help alleviate the problems experienced by children with PNS and improve their quality of life.

## Subjects and Methods

### Study Subjects

A total of 246 children with PNS who were hospitalized in the pediatric nephrology ward at the ShengJing Hospital of the China Medical University from March 2019 to October 2020 were selected as study subjects. The parents of 231 of these patients agreed to participate in the research (participation rate = 93.9%). The diagnostic criteria for PNS were as follows (4): (1) 24-h proteinuria >50 mg/kg, (2) serum albumin level <30 g/L, (3) serum cholesterol level >5.7 mmol/L, and (4) varying degrees of edema. All the children included in the study met these diagnostic criteria, and their parents voluntarily cooperated with the survey.

### Survey Methods

A pre-established WeChat communication management group was used for the participants, and an online questionnaire was shared with the parents after joining the group and was completed on-site, with the assistance of the nurses to ensure its accurate completion. The survey was specifically designed for the study and was suitable for the children's age groups. The survey included a section to gather information on the general characteristics of the patient; the Mandarin Chinese version of the Pediatric Quality of Life Inventory 4.0 (PedsQL™ 4.0) generic core scales (GCS) for the age groups 2–4, 5–7, 9–12, and 13–18 year; and a knowledge needs assessment and psychological survey for parents of children with PNS. Before filling out the questionnaire, the nurse explained the questionnaire and instructed the subject on how to fill it out correctly.

The PedQL™ 4.0-GCS is a multi-dimensional tool designed to evaluate the condition of healthy children and children with chronic diseases and consists of child self-assessment and parent report scales (5, 6). However, in PedQL™ 4.0-GCS, the parent report scale is only available for children aged 2–4 year. For consistency, we adopted a parent report scale for each age group in this study.

The PedQL™ 4.0-GCS encompasses 23 items that include physical and psychological characteristics. Physical functioning consists of eight items, and psychological functioning is split into three areas, i.e., emotional, social, and school functioning, each consisting of five items. PedQL™ 4.0-GCS measures the frequency of occurrence of each item within the past month. The results reported by parents were divided into five scales based on the degree of difficulty experienced: 0 = no problem (100 points), 1 = rarely a problem (75 points), 2 = sometimes a problem (50 points), 3 = often a problem (25 points), and 4 = always a problem (0 points). A final score was calculated based on the average score for each item. The higher the score, the better the quality of life.

### Statistical Methods

The data were statistically analyzed using the SPSS 23.0 software. Countable data were expressed as frequencies and percentages. The data did not satisfy the normal distribution, and the rank-sum test was adopted for data analysis. *P* < 0.05 was considered statistically significant.

## Results

### General Characteristics

A total of 231 children with PNS participated in the survey including 152 males and 79 females. The average age of the children was 6.55±3.71 year, with 91.80% of the children aged 2–12 year. In 91.30% of cases, the disease duration was <5 year, and approximately 51% of families lived in urban areas. The general characteristics of the study subjects are summarized in Table 1.

**Table 1:** The general characteristics in the children with nephrotic syndrome.

**Project**	***n*** **(%)**	**Project**	***n*** **(%)**
**Gender**		**Disease duration (year)**	
Male	152 (65.8)	<1	125 (54.1)
Female	79 (34.20)	1–3	59 (25.50)
		3–5	27 (11.70)
		>5	20 (8.70)
**Age (year)**		**Location of family**	
2–4	93 (40.30)	Urban	118 (51.10)
5–7	62 (26.8)	Rural	78 (33.80)
8–12	57 (24.70)	Rural-urban intersection	35 (15.20)
13–18	19 (8.20)		

### Results of the PedsQL™

#### Overall Quality of Life Scores of Children With PNS

Total quality of life scores and scores for each physical and psychological area were significantly lower for children with PNS than for healthy children (7). The average quality of life score was 79.60 ± 14.02 points, and only 6.49% of children achieved a perfect score, with 90.47% of children experiencing mild to moderate negative effects (severe effects were rare). Psychological functioning was the area most impacted by PNS, with only 8.23% of children scoring 100 points in this area. The school functioning score was the lowest, which is consistent with the results reported by Selewski et al. (8) who found that children with PNS had significantly lower school functioning scores than healthy children. Social and emotional functioning were less severely impacted, with >30% of children with PNS scoring maximum points in these areas, and the majority of the remaining children scoring 50–99 points, indicating only a slight to moderate effect. The detailed scores are shown in Table 2.

**Table 2:** The survey results by PedsQL™ 4.0 Pediatric Quality of Life in children with PNS.

**Items**	**Average score**	**Proportion of each score(%)**			
		**0–50** **points**	**50.1–75** **points**	**75.1–99.9** **points**	**100** **points**
Physical functioning scale	82.69 ±16.09	5.19	23.81	54.98	16.02
Psychological functioning scale	77.80 ± 14.90	4.76	40.69	46.32	8.23
Social functioning scale	87.06 ± 14.97	2.16	24.68	33.33	39.83
School functioning scale	67.11 ± 26.33	25.11	37.23	19.48	18.18
Emotional functioning scale	76.65 ± 17.64	8.23	43.29	24.68	37.23
Total score	79.60 ± 14.02	3.03	34.68	55.84	6.49

#### Detailed Item Scores Relating to the Quality of Life of Children With PNS

On the physical functioning scale, 83% of the children had no problems with light physical activities, such as walking more than 100 m, but more than one-third experienced difficulties with moderately heavy or heavy physical activities, such as running and other sporting activities, taking a bath or shower, and carrying out household chores, and more than 50% said they always felt pain and fatigue. Social functioning was the only of the three areas of psychological functioning in which the children achieved high scores; however, more than one-third reported problems getting along with other teenagers and making friends and 34% were teased by others. On the school functioning scale, 83% reported being chronically absent or dropping out of school due to the disease, and most children reported problems with attention and memory. More than half of the children described feeling angry, sad, scared, or fearful on the emotional functioning scale; 45% had sleeping problems; and 44% were worried about accidents. The detailed item scores are shown in Table 3.

**Table 3:** The detailed item scores for the quality of life in children with PNS.

**Item**		**No problem**	**Had difficulties**	**Serious problems**
		**100 points (%)**	**0–75 points (%)**	**0–50 points (%)**
Physical functioning scale	(1) Walk more than 100 meters	83	17	6
	(2) Run	69	31	19
	(3) Do sports activity or exercise	57	43	27
	(4) Lift something heavy	60	40	22
	(5) Take a bath or shower by oneself	66	34	16
	(6) Do chores around the house	56	44	20
	(7) Feeling pain	46	54	31
	(8) Feeling fatigue	40	60	41
Emotional functioning scale	(1) I feel afraid or scared	40	60	38
	(2) I feel sad or blue	49	51	28
	(3) I feel angry	31	69	52
	(4) I have troubled sleeping	55	45	23
	(5) I worry about what will happen to me	56	44	24
Social functioning scale	(1) I have trouble getting along with other kids	63	37	17
	(2) Other kids do not want to be my friends	63	37	13
	(3) Other kids tease me	66	34	10
	(4) I cannot do things that other kids my age can do	68	32	16
	(5) It is hard to keep up when I play with other kids	65	35	15
School functioning scale	(1) It is hard to pay attention in class	46	54	30
	(2) I forget things	49	51	25
	(3) I have trouble keeping up with my schoolwork	52	48	24
	(4) I do not attend school due to illness	23	77	64
	(5) Not attending school due to medical appointments or hospital visits	17	83	72

#### The Impact of Family, Age, Gender, and Disease Duration on The Quality of Life of Children With PNS

Families were categorized into one of three groups according to their location: urban, rural, or urban–rural intersection. There were no significant differences between the groups based on the total quality of life score or the psychological, physical, emotional, social, or school functioning scales (*p* > 0.05). There were no significant differences in terms of social and school functioning based on age (*p* > 0.05). However, there were significant differences in the total score and psychological, physical, and emotional functioning scales between the different age groups (*P* < 0.05), with children aged 5–7 year achieving the lowest scores. Children aged 2–4 year scored highest on the physical functioning scale, which may be correlated with the short duration of the disease. The school functioning score was significantly higher for males than females (*P* < 0.05), but no other significant differences were found based on gender (*p* > 0.05).

In terms of disease duration, total quality of life scores were highest within 1 year of diagnosis (82.44 ± 12.20 points), and significant differences in physical, social, emotional, and school functioning scales were found based on disease duration (*P* < 0.05). The impact on physical functioning 1–3 year after diagnosis was slight but became more apparent after this point, and the impact on the psychological functioning scale was minor during the first year after diagnosis but became more pronounced after the first year had passed. The lowest social, school, and emotional functioning scores were found in children with a disease duration of 1–3 year. We speculate that this is due to an insufficient understanding and incomplete acceptance of the disease by both children and parents within 3 years of onset. As the duration of the disease increases, children gradually adapt to a life of medication, dietary changes, and daily urine protein tests, and family members gradually learn and understand more about PNS. Therefore, the psychological and physiological impact of PNS on the children and their family members gradually decreases. Detailed information on these findings is displayed in Table 4.

**Table 4:** The effect of family, age group, gender, and course of disease on the item scores in children with PNS.

		**Physical** **functioning** **scale**	**Psychological** **functioning** **scale**	**Emotional** **functioning** **scale**	**Social** **functioning** **scale**	**School** **functioning** **scale**	**Total** **score**
Location of family	Urban (*n* = 118)	84.14 ± 14.04	78.57 ± 13.43	78.69 ± 16.85	88.47 ± 13.37	64.60 ± 27.36	80.65 ± 12.25
	Rural (*n* = 78)	80.93 ± 18.27	77.29 ± 16.31	73.72 ± 18.47	85.96 ± 16.46	71.13 ± 24.37	78.62 ± 15.68
	Rural-urban intersection (*n* = 35)	81.70 ± 17.40	76.32 ± 16.54	76.29 ± 17.88	84.71 ± 16.49	66.57 ± 26.64	78.25 ± 15.74
	*P*	0.69	0.80	0.16	0.45	0.20	0.77
Age	2–4 years (*n* = 93)	88.01 ± 13.40	80.71 ± 14.67	80.11 ± 17.38	90.75 ± 11.20	65.14 ± 33.59	83.50 ± 12.90
	5–7 years (*n* = 62)	78.33 ± 17.20	72.29 ± 14.33	68.47 ± 16.95	83.31 ± 16.55	64.65 ± 21.72	74.40 ± 13.89
	8–12 years (*n* = 57)	78.67 ± 18.08	78.92 ± 15.06	78.95 ± 16.92	85.96 ± 16.32	71.84 ± 19.29	78.83 ± 15.18
	13–18 years (*n* = 19)	82.90 ± 10.49	78.16 ± 13.52	79.47 ± 15.71	84.47 ± 18.33	70.52 ± 14.52	79.80 ± 10.84
	*P*	0.00	0.01	0.00	0.08	0.45	0.00
Gender	Male (*n* = 152)	83.78 ± 14.61	78.84 ± 14.82	77.50 ± 17.79	87.24 ± 15.06	69.61 ± 25.42	80.66 ± 13.63
	Female (*n* = 79)	80.58 ± 18.54	75.79 ± 14.94	75.00 ± 17.32	86.71 ± 14.89	62.30 ± 27.51	77.57 ± 14.60
	*P*	0.37	0.17	0.39	0.60	0.04	0.12
Course	<1 year (*n* = 79)	85.68 ± 13.27	80.52 ± 13.68	77.36 ± 16.75	90.72 ± 12.21	70.83 ± 25.99	82.44 ± 12.20
	1–3 years (*n* = 79)	80.83 ± 18.57	72.86 ± 16.74	75.85 ± 18.22	82.29 ± 17.08	56.13 ± 30.5	75.78 ± 16.23
	3–5 years (*n* = 79)	76.85 ± 18.81	76.86 ± 12.44	73.89 ± 19.87	84.26 ± 15.17	72.1 ± 16.06	76.87 ± 13.4
	>5 years (*n* = 79)	77.35 ± 17.32	76.58 ± 16.47	78.25 ± 19.01	82 ± 18.38	69.5 ± 16.77	76.84 ± 15.41
	*P*	0.03	0.02	0.86	0.00	0.01	0.02

### Knowledge Needs and Psychological Problems in Parents of Children With PNS

Parents identified the following as areas in which they required more information: etiology (78%), prognosis (75%), diet (52%), children's ability to attend school (52%), treatment (47%), recurrence rate (41%), other immunosuppressive agents (20%), and costs (19%). Parents reported the following psychological issues relating to their child's health: anxiety (59%), sadness (44%), fear (43%), and depression (40%). Several parents also mentioned experiencing feelings of self-blame.

## Discussion

PNS is a common chronic glomerular disease in children characterized by a prolonged duration and recurrent nature and requiring the long-term use of steroids and other immunosuppressive agents. PNS has a significant negative impact on the quality of life, social activities, psychological status, growth, and development of children. Understanding the physiological, psychological, and social problems of children with PNS and the knowledge needs of their parents can be beneficial in helping to improve the quality of management of PNS and other chronic diseases in order to enhance the patients' quality of life.

In a previous study, the PedQL™ 4.0-GCS was used to evaluate the quality of life of children with diabetes mellitus, and the total score was found to be lower than that of healthy children (2). Similarly, the scales have been widely utilized in the evaluation of other pediatric diseases, such as diabetes (9), cancer (10), and systemic lupus erythematosus (SLE) (11), and the results suggest that the quality of life of children with chronic diseases is generally low (12).

In this study, we used PedQL™ 4.0-GCS to investigate the quality of life of 231 children with PNS and the disease awareness of their parents. We found that the influence of PNS on the physical state of the children was universal, and the psychological impact was obvious in the children and their parents. Children aged 5–7 year experienced the most severe problems, and the psychological issues faced by children with PNS and their parents within 3 years of diagnosis were intense and problematic. After 3 years, most subjects' psychological symptoms gradually lessened due to their familiarity with the routine of taking medication, ability to adapt to a low-fat, high-quality protein diet and daily urine protein tests, and improved understanding of the disease.

Approximately 84% of the children in this study experienced a negative impact of PNS on their physical functioning, which affected their quality of life. These symptoms worsen as the disease duration increases due to the impact of long-term steroid or other immunosuppressive agent therapy. In a previous study of children with SLE, Moorthy et al. (11) showed that the administration of steroids and immunosuppressive agents affected the quality of life, leading to osteoporosis and the significant inhibition of growth and development, which in turn led to an inability to participate in physical activities, such as running and lifting heavy objects. We found that the social functioning of the children with PNS in the study was lower than that of healthy children. Due to frequent medical visits, children with PNS either dropped out of school entirely or were absent for long periods, were unable to participate in some group activities, lacked normal social communication, and were teased by others, resulting in a reluctance to return to school or engage with other children, thus further reducing their social functioning. Previous research has shown that absence from school has a negative impact on children's quality of life (13).

Across all demographic characteristics in this study, the school functioning scores were always among the lowest. Some of the factors contributing to these low scores are the need for frequent medical treatment in hospital, which results in absence from school and poor academic performance; physical discomfort, which affects learning ability; and the low expectations of parents and teachers for the child's academic performance. We also found the emotional functioning of children with PNS to be lower than that of healthy children. Repeated medical visits, long-term medication, somatic discomfort, and changes in appearance caused by steroid therapy caused feelings of sadness, anger, and fear, and during medical treatment, many parents described their children as silent, precocious, or anxious. The quality of life scores of females were found to be lower than those of males, which is consistent with the results of previous studies (14–16). The psychological domain consists of social, emotional, and school functioning, and problems in any of these areas could affect the psychological wellbeing of children with PNS.

The survey of the needs of the parents revealed that their primary concern (78% of parents) was in understanding the cause of the disease, which may be related to the feelings of self-blame expressed by some of the parents. The parents typically presented with a heavy psychological burden, including anxiety, sadness, irritability, and other adverse emotions. Such feelings may also indirectly affect the child's psychological state, which can further negatively impact on their quality of life (17). Rüth et al. (1) showed that parents' (especially mothers') negative emotions severely negatively impacted the quality of life and psychological wellbeing of children with PNS, which may cause a further increase in the negative emotions of the parents, forming a vicious cycle (18). Pediatricians and nurses want to offer long-term standardized treatment to children with PNS through a comprehensive management protocol to improve their quality of life (19). However, insufficient information on PNS makes comprehensive disease management difficult; the assessment, monitoring, and management of children with PNS continues to be problematic for parents and pediatricians; and a lack of understanding of PNS among educational institutions affects the ability of children with PNS to effectively return to society.

In summary, we investigated the quality of life of 231 children with PNS and the disease awareness of their parents. We found that PNS has a significant negative impact on the physical and psychological wellbeing of children and their parents. We hope that the present survey draws attention to the issues faced by children with chronic diseases and their families.

The first limitation of this study is that although it investigated enough cases to describe the associations between the variables, it lacked a control group. Second, the group in this study was not representative of populations in other cities, provinces, or countries. These limitations should be addressed in future studies.

Children with PNS and their parents require comprehensive management and care from doctors, nurses, dieticians, psychotherapists, educational institutions, and the wider public to reduce their psychological burden, improve treatment compliance, increase their confidence in returning to society, and ultimately, improve their quality of life.

## Data Availability Statement

The original contributions presented in the study are included in the article/supplementary material, further inquiries can be directed to the corresponding author.

## Ethics Statement

The studies involving human participants were reviewed and approved by Ethics Committee of Shengjing Hospital of China Medical University (2019PS096J). Written informed consent to participate in this study was provided by the participants' legal guardian/next of kin.

## Author Contributions

NL and JH: acquisition of data. NL and YD: analysis and interpretation of the data and critical revision of the manuscript for intellectual content. NL: statistical analysis. YD: obtaining financing. NL,TF, and YD: writing of the manuscript, conception, and design of the research. All authors read and approved the final draft.

## Funding

Cultivation Discipline Project Support of China Medical University (No. 3110118042).

## Conflict of Interest

The authors declare that the research was conducted in the absence of any commercial or financial relationships that could be construed as a potential conflict of interest.

## Publisher's Note

All claims expressed in this article are solely those of the authors and do not necessarily represent those of their affiliated organizations, or those of the publisher, the editors and the reviewers. Any product that may be evaluated in this article, or claim that may be made by its manufacturer, is not guaranteed or endorsed by the publisher.
